# Supplementary material for: Computed tomography reveals hip dysplasia in the extinct Pleistocene saber-tooth cat Smilodon
Source: Sci Rep. 2021 Oct 28;11:21271. doi: 10.1038/s41598-021-99853-1 (PMC8553773; doi:10.1038/s41598-021-99853-1)
Supplement: Supplementary file 1 — Supplementary Information. [file 41598_2021_99853_MOESM1_ESM.pdf]

Computed tomography reveals hip dysplasia in the extinct Pleistocene saber-tooth cat *Smilodon*

Mairin A. Balisi, Abhinav K. Sharma, Carrie M. Howard, Christopher A. Shaw,

Robert Klapper, Emily L. Lindsey

Supplementary Information

Supplementary File on Dryad (doi:10.6071/M3S09M)

Supplementary Videos on Dryad (doi:10.6071/M3S09M)

Supplementary Data on Dryad (doi:10.6071/M3S09M)

Supplementary Text (in this document)

Supplementary References (in this document)

Supplementary Figures (in this document)

Supplementary File S1. Three-dimensional PDF of the pathological pelvis (LACMHC 131) and femur (LACMHC 6963).

Supplementary Video S1. A video movie of a structured light surface scan of LACMHC 131, the pathological pelvis belonging to *Smilodon fatalis*. Movie created in Adobe Photoshop CC by Carrie Howard.

Supplementary Video S2. A video scrolling anteroposteriorly through LACMHC 131, the pathological pelvis belonging to *Smilodon fatalis*. Ventral is at top. Video created by Carrie Howard from CT scans generated at the S. Mark Taper Foundation Imaging Center at Cedars Sinai Medical Group.

Supplementary Data S1. Compressed zip file containing the full computed tomography scan of LACMHC 131, the pathological pelvis.

Supplementary Data S2. Compressed zip file containing the full computed tomography scan of LACMHC 6963, the pathological femur.

Supplementary Data S3. Compressed zip file containing the full computed tomography scan of LACMHC K-3232, the non-pathological right femur.

Supplementary Data S4. CSV file containing basic morphometric data for comparative *Smilodon fatalis* pelvis specimens, both pathological and non-pathological. LACM = Los Angeles County [Natural History] Museum; HC = Hancock Collection.

Supplementary Data S5. CSV file containing basic morphometric data for comparative *Smilodon fatalis* femur specimens, all non-pathological. LACM = Los Angeles County [Natural History] Museum; HC = Hancock Collection.

Supplementary Text

Callus formation defines secondary healing of bones following a fracture, which is characterized by a soft cartilage intermediate, or callus, that bridges the two fracture fragments and provides early stability to the fracture site[1]. Secondary bone healing occurs as a result of motion at the fracture site, as cells respond to mechanical stresses across the fracture fragments, ultimately resulting in the recruitment of stem cells that deposit bone matrix[2]. Secondary bone healing entails four sequential steps, including inflammation and hematoma formation, fibrocartilaginous (soft callus) formation, bony (hard callus) formation, and finally, bone remodeling (Supplementary Fig. S11). Hematoma formation occurs immediately after a fracture and is the result of ruptured vessels and blood supply surrounding the fracture[1,2]. Within approximately two weeks in humans, fibrocartilaginous soft callus forms due to release of various cytokines that promote angiogenesis, formation of fibrin-rich granulation tissue, and chondrogenesis, which is the foundation for the growth of new bone[1,2]. These chondrocytes undergo endochondral ossification and differentiate further into bone-forming cells, including osteoblasts and osteoclasts, resulting in the calcification of the soft callus[2]. In humans, hard callus forms between six and 12 weeks following a fracture and undergoes repeated remodeling with continued migration of osteoblasts and osteoclasts from months to years onward, often up to six to nine years[1,2] (Supplementary Fig. S11). This process results in the regeneration of typical bone architecture.

The bony changes observed in LACMHC 131 and 6963 are less consistent with this process of secondary bone healing and callus formation. The sclerosis and reactive bone formation observed in the specimen are diffusely located around the acetabulum

and hip as well as the femoral head, as opposed to singularly located around a potential fracture site as would be seen following an acute injury. Additionally, the anatomical distortions of the underdeveloped acetabulum in conjunction with the aspherical right femoral head more fittingly characterizes chronic remodeling due to mechanical instability and repeated subluxation events in the setting of a lack of concentric reduction of the femoral head in the shallow acetabulum. The changes observed in both components of the hip joint are more typically observed in dysplastic hips.

Supplementary References

1. Sheen, J. R. & Garla, V. V. Fracture Healing Overview. in *StatPearls* (StatPearls Publishing, 2021).
2. Ghiasi, M. S., Chen, J., Vaziri, A., Rodriguez, E. K. & Nazarian, A. Bone fracture healing in mechanobiological modeling: A review of principles and methods. *Bone Rep* **6**, 87–100 (2017).

Supplementary Figures

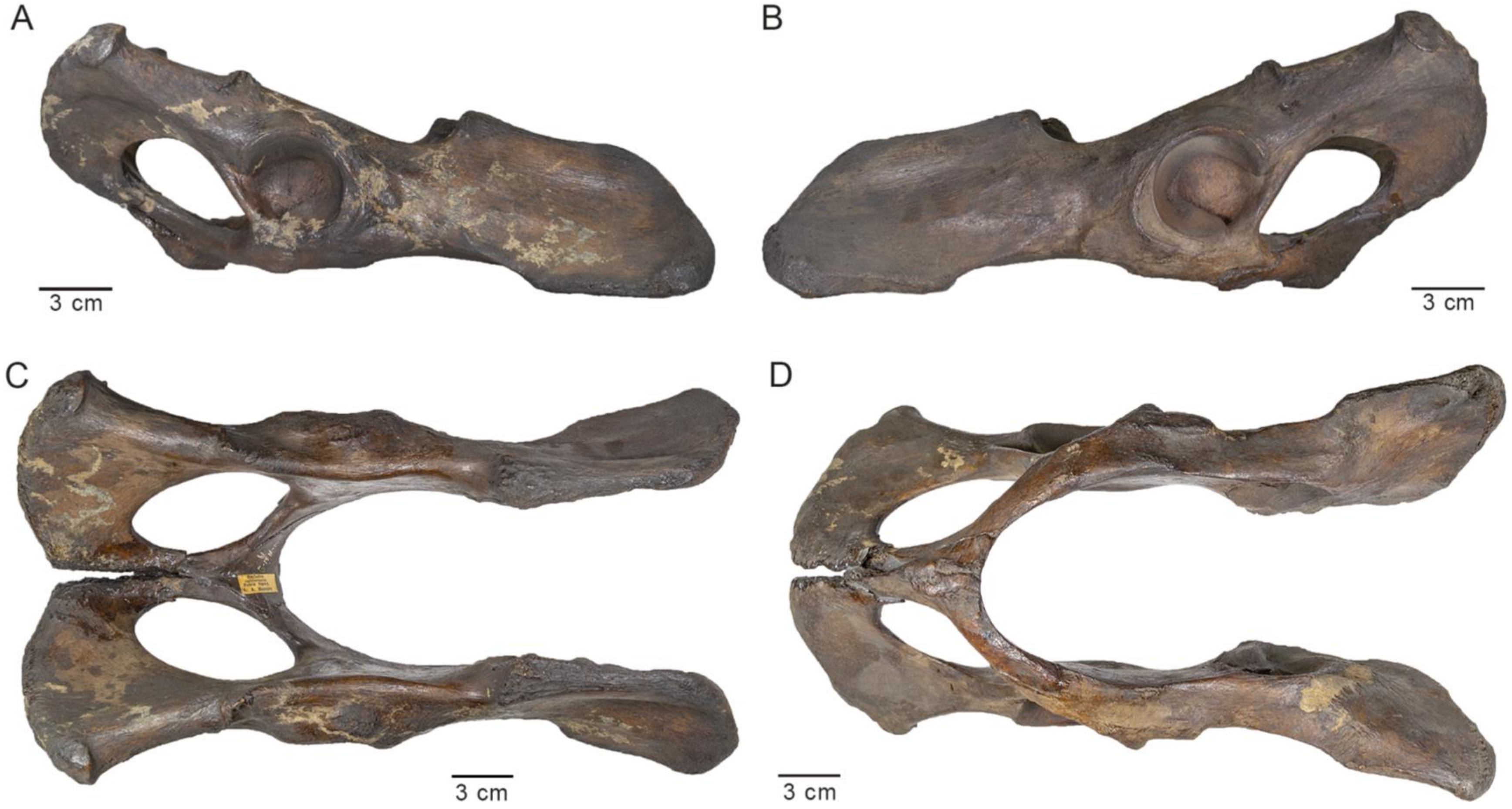

Supplementary Fig. S1. Photographs of LACMHC K-2584, a non-pathological pelvis belonging to *Smilodon fatalis*. **(A)** Lateral view of right side; anterodorsal end to the right. **(B)** Lateral view of left side; anterodorsal end to the left. **(C)** Dorsal and **(D)** ventral views; anterior end to the right.

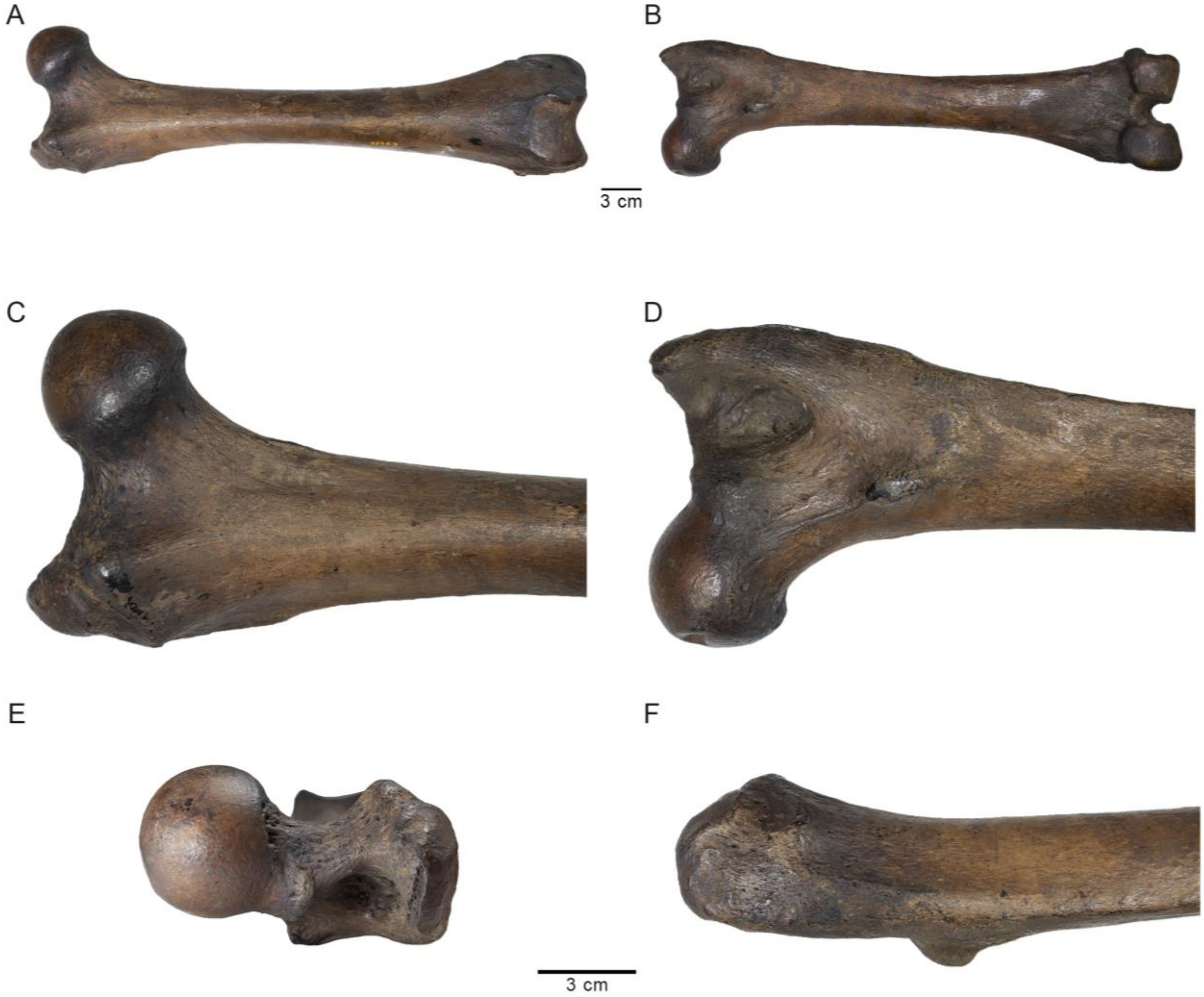

Supplementary Fig. S2. Photographs of LACMHC K-3232, a non-pathological right femur belonging to *Smilodon fatalis*. **(A)** Anterior and **(B)** posterior views of full femur; proximal end on the left. **(C)** Anterior and **(D)** posterior close-up views of the proximal end, including the spherical femoral head, greater trochanter, and lesser trochanter. **(E)** Dorsal close-up view of the femoral head, greater trochanter, and lesser trochanter in lower center background. **(F)** Lateral close-up view of the greater trochanter and lesser trochanter (lower center). The upper scale bar refers to A and B, and the lower scale bar refers to C, D, E, and F.

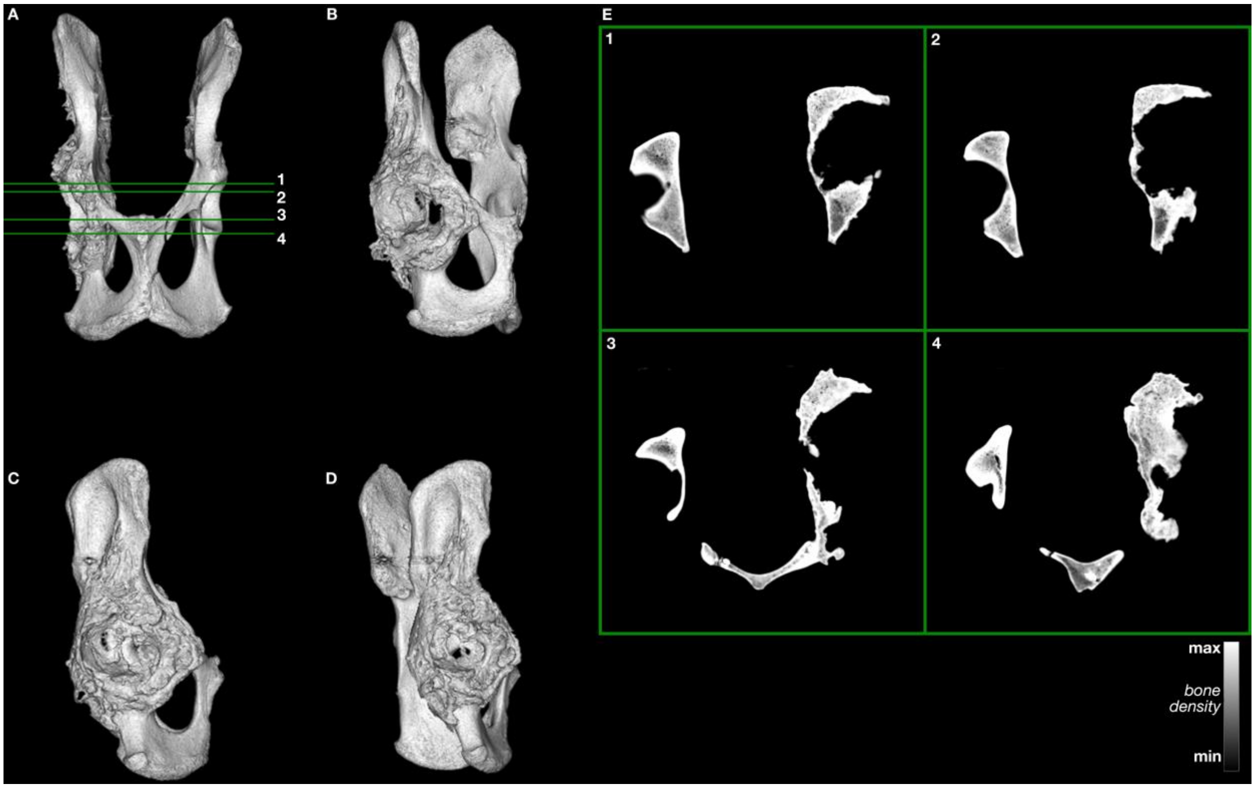

Supplementary Fig. S3. Greyscale images of bone sections of the pathological pelvis, LACMHC 131. Figure is the same as Fig. 3 in the Main Text but without the density color filter.

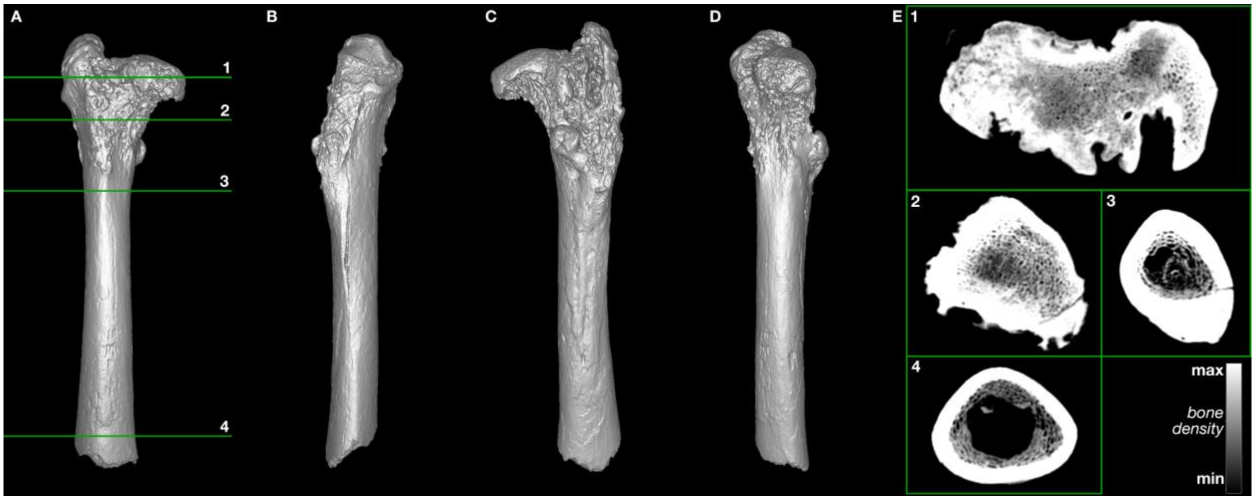

Supplementary Fig. S4. Greyscale images of bone sections of the pathological right femur, LACMHC 6963. Figure is the same as Fig. 4 in the Main Text but without the density color filter.

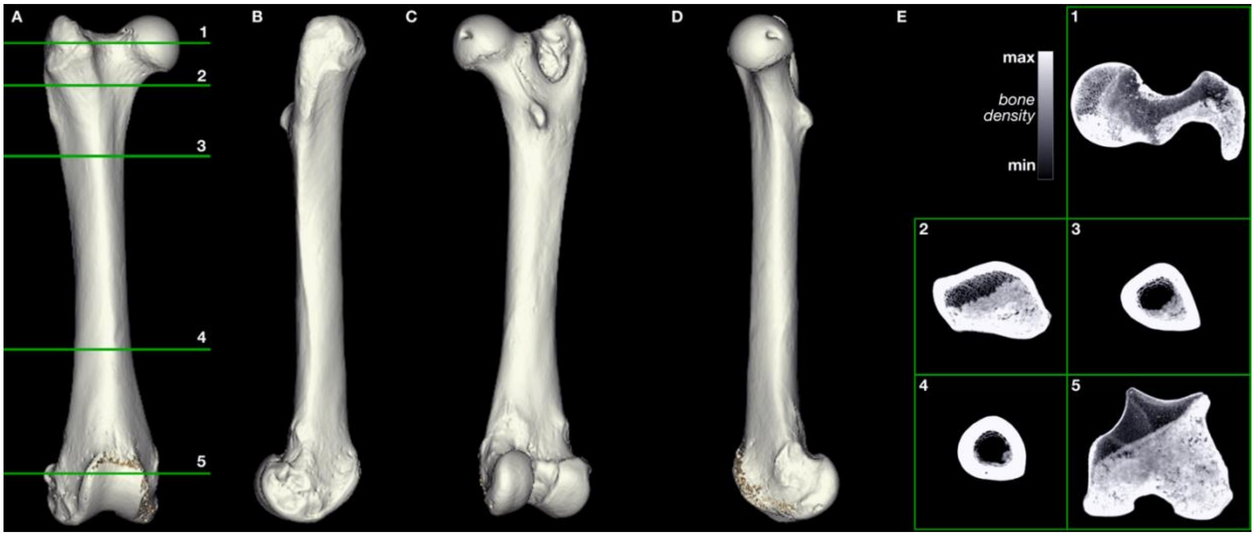

Supplementary Fig. S5. Greyscale images of bone sections of a non-pathological right femur, LACMHC K-3232. Figure is the same as Fig. 5 in the Main Text but without the density color filter.

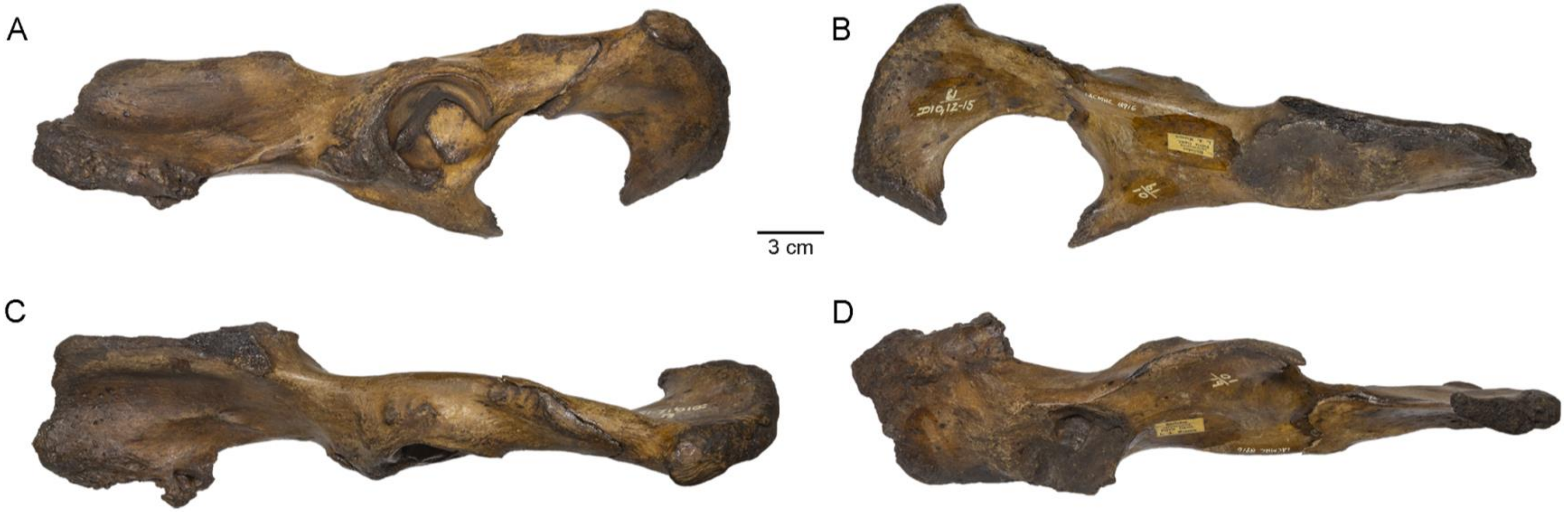

Supplementary Fig. S6. Photographs of LACMHC 8916, a pathological left innominate of *Smilodon fatalis* from Pit 61/67 exhibiting potential signs of dysplasia, in particular heavy exostoses and possible traction injuries in the origins of the *M. rectus femoris* and *M. iliacus*. **(A)** Lateral view; anterodorsal end to the left. **(B)** Medial view; anterodorsal end to the right. **(C)** Dorsal and **(D)** ventral views; anterior end to the right.

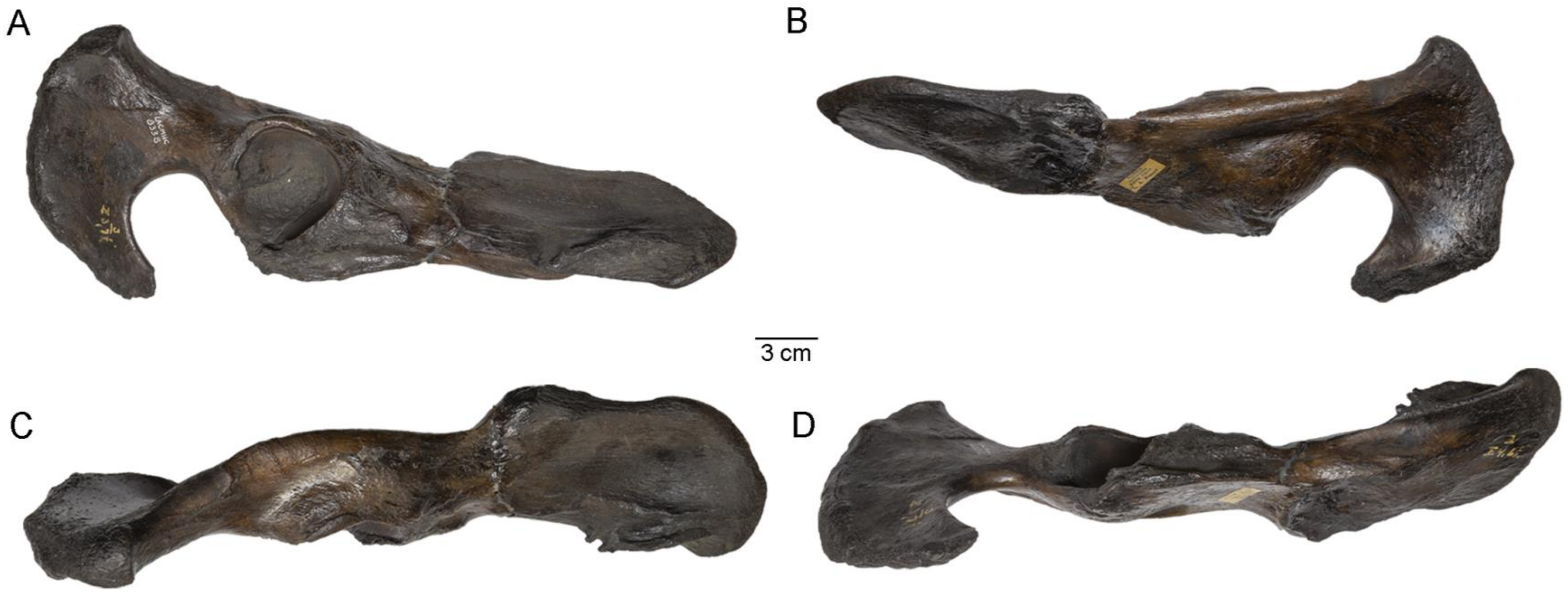

Supplementary Fig. S7. Photographs of LACMHC 8338, a pathological right innominate of *Smilodon fatalis* from Pit 3 exhibiting potential signs of dysplasia. Deepening of the acetabulum has caused a bulge in the medial wall, likely impacting gait and weight-bearing, and the origins of the *M. iliacus* and *M. gluteus medius* exhibit traction injuries. **(A)** Lateral view; anterodorsal end to the right. **(B)** Medial view; anterodorsal end to the left. **(C)** Dorsal and **(D)** ventral views; anterior end to the right.

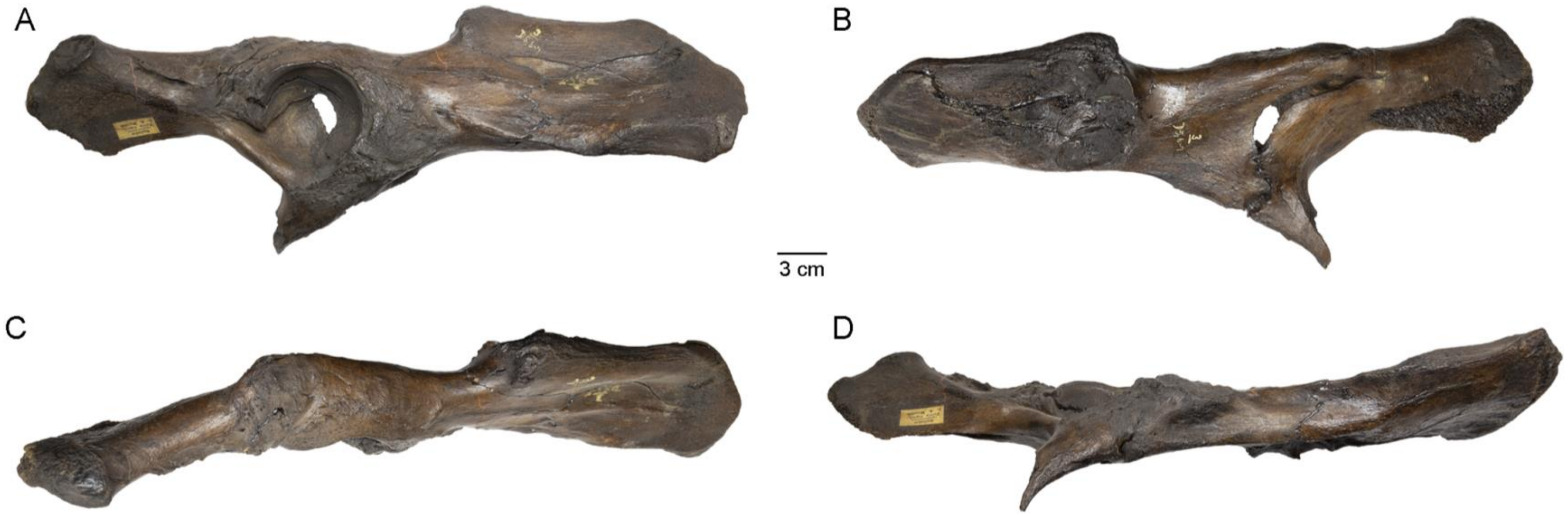

Supplementary Fig. S8. Photographs of LACMHC 8342, a pathological right innominate of *Smilodon fatalis* from Pit 3 exhibiting potential signs of dysplasia. A healed fracture crosses the acetabulum; the associated callus build-up has united in a line involving both the pubis and the ischium at an oblique angle. The acetabular joint is intact and smooth, however, and the matching femur would be of great interest if found. **(A)** Lateral view; anterodorsal end to the right. **(B)** Medial view; anterodorsal end to the left. **(C)** Dorsal and **(D)** ventral views; anterior end to the right.

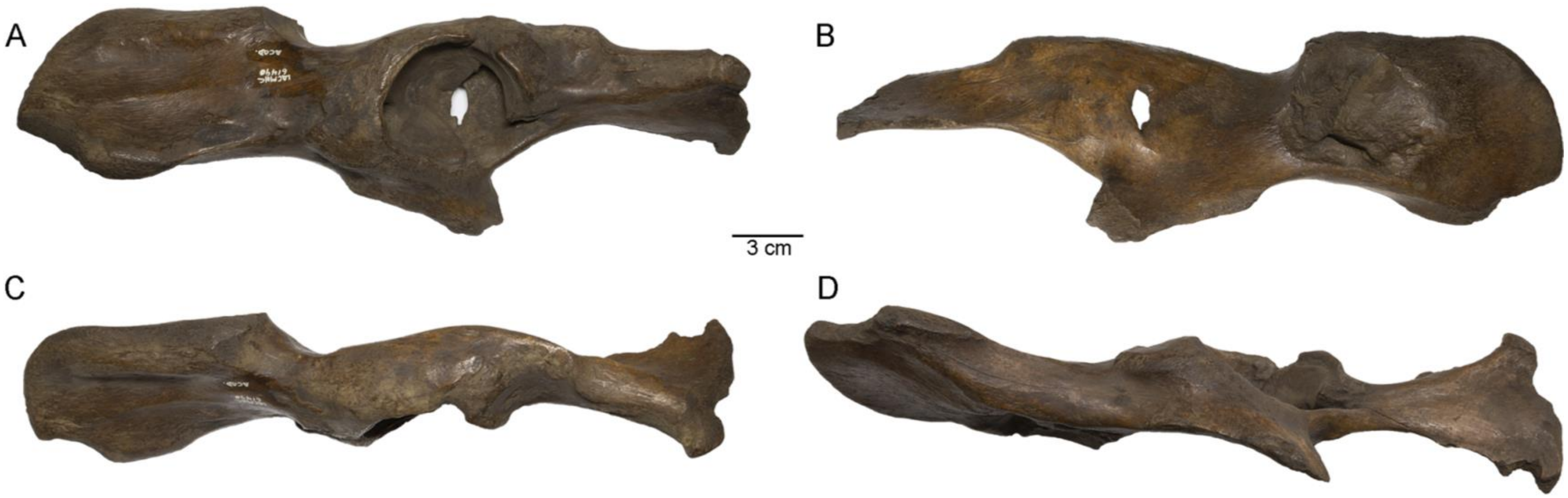

Supplementary Fig. S9. Photographs of LACMHC 61490, a pathological left innominate of *Smilodon fatalis* from Pit “Academy” exhibiting potential signs of dysplasia. The posterior wall of the acetabulum is wrinkled deeply, and the anterodorsal aspect exhibits an apparent incompletely healed fracture. CT imaging of the internal bone structure would aid in identifying etiology. **(A)** Lateral view; anterodorsal end to the left. **(B)** Medial view; anterodorsal end to the right. **(C)** Dorsal and **(D)** ventral views; anterior end to the right.

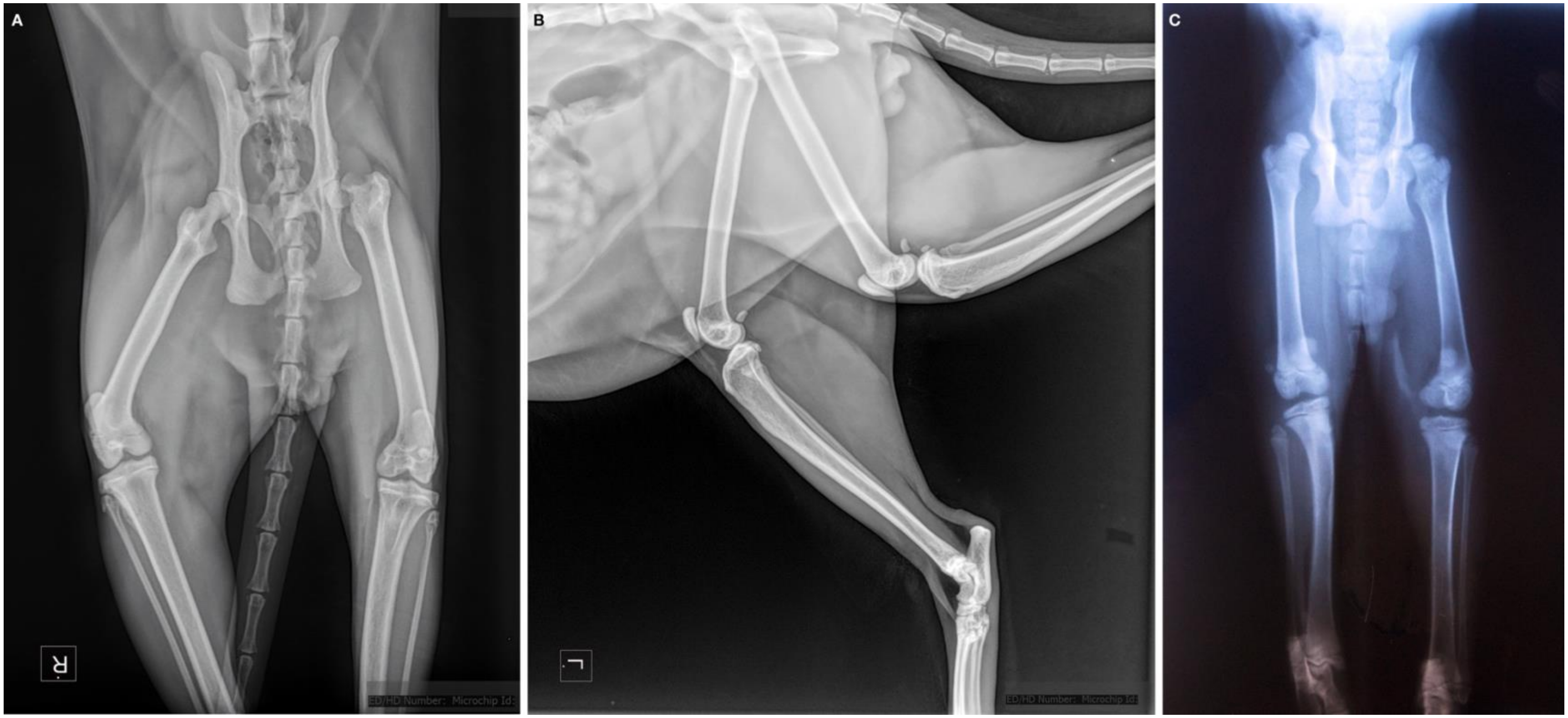

Supplementary Fig. S10. X-ray images of two domestic cats, both of which exhibit the radiographic hallmarks of hip dysplasia. **(A)** Ventrodorsal and **(B)** lateral radiographs of the pelvis and hip joints of an individual demonstrating severe left hip dysplasia. The hypoplastic and shallow left acetabulum, characteristic of dysplasia, has resulted in significant femoral head deformity, subluxation, and dislocation of the hip joint. Relative to the right hip, there is evidence of chronic remodeling and degenerative arthritic changes in the left acetabulum and femoral head, as well as shortening of the left leg and asymmetry between the two extremities. Additionally, the left femoral head is flattened and severely anatomically distorted. The lateral view demonstrates asymmetry in the left and right hip joints due to the dorsally dislocated left hip. **(C)** Ventrodorsal view of the pelvis and hip joints of another individual demonstrating signs of right hip dysplasia. The shallow right acetabulum does not provide adequate coverage for the right femoral head to fit concentrically, resulting in hip instability. The right femoral head is proximally dislocated, creating a high-riding hip. (A-B) courtesy of Carrie M. Howard, (C) courtesy of Karin A. Rice.

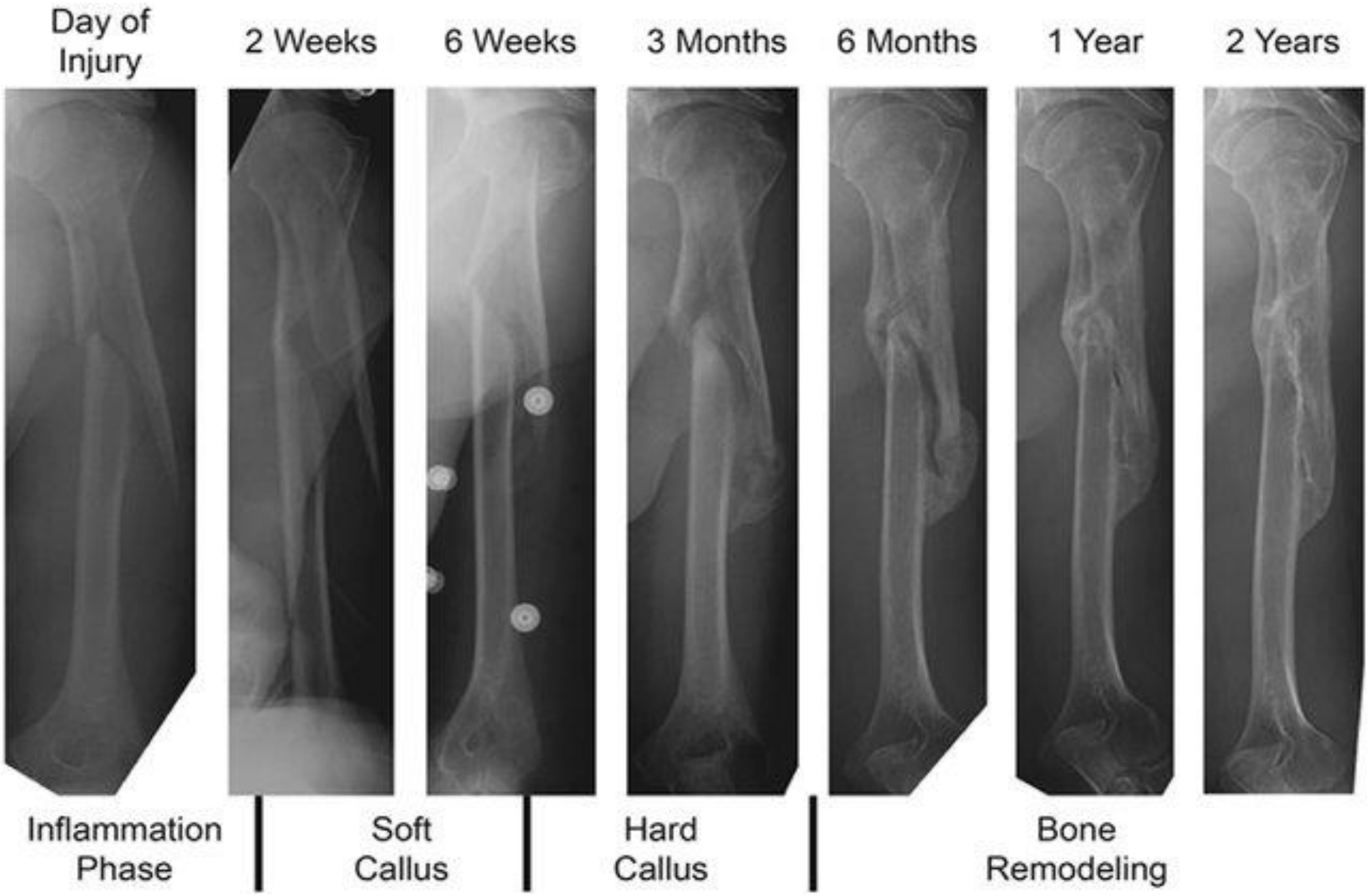

Supplementary Fig. S11. Clinical example of secondary bone healing in a human humerus fracture. Within two weeks of fracture, the formation of soft callus occurs. As the fracture heals, the callus becomes larger and eventually develops into hard callus. Bone remodeling subsequently begins months after injury and continues for years. Adapted with written permission from: Ghiasi MS, Chen J, Vaziri A, Rodriguez EK, Nazarian A. Bone fracture healing in mechanobiological modeling: A review of principles and methods. *Bone Rep.* 2017 Mar 16;6:87-100. doi: 10.1016/j.bonr.2017.03.002. PMID: 28377988; PMCID: PMC5365304.
